# The complete mitochondrial genome of *Nycteribia parvula* Speiser, 1901 (Diptera, Nycteribiidae)

**DOI:** 10.1080/23802359.2023.2169573

**Published:** 2023-02-21

**Authors:** Jinting Yang, Xiaobin Huang, Huijuan Yang, Yujuan Wang, Xianzheng Zhang, Xiaoyan Zheng

**Affiliations:** a Institute of Pathogens and Vectors, Yunnan Provincial Key Laboratory for Zoonosis Control and Prevention, Dali University, Dali, China; b Jilin Provincial Key Laboratory of Animal Resource Conservation and Utilization, Northeast Normal University, Changchun, China

**Keywords:** Mitochondrial genome, *Nycteribia parvula*, Nycteribiidae, bat fly

## Abstract

Species of the family Nycteribiidae are blood-sucking ectoparasites that parasitize bats. To further enrich the molecular data of species in the family Nycteribiidae, the complete mitochondrial genome of *Nycteribia parvula* was sequenced for the first time in this study. The complete mitochondrial genome of *N. parvula* is 16,060 base pairs (bp) in size, including 13 protein-coding genes (PCGs), 22 transfer RNA genes, two ribosomal RNA genes, and a control region. The nucleotide contents of A, T, G, and C are respectively 40.86%, 42.19%, 6.51%, and 10.44%. The phylogenetic analysis based on 13 PCGs supports the monophyly of the family Nycteribiidae, and *N. parvula* is the closest relative to *Phthiridium szechuanum*.

## Introduction

The family Nycteribiidae belongs to the superfamily Hippoboscoidea of the Diptera, which are called bat flies together with the family Streblidae. The families Nycteribiidae and Streblidae are the obligate ectoparasites of bats, with both sexes feeding on blood and residing in the hair or on the wing membranes (Poinar and Brown 2012). The morphological traits of the Nycteribiidae are strikingly specialized compared with other flies, including lack of wings, reduced head and eyes, a dorsoventrally flattened thorax, dorsally inserted legs, and a spider-like appearance (Hosokawa et al. 2012).

Through molecular research, many scholars have found a variety of pathogens inside them, such as Bartonella (Low et al. 2022), Wolbachia (Tseng et al. 2019), Haemosporidian (Sándor et al. 2021), Dengue Virus (Abundes-Gallegos et al. 2018), orthobunyavirus (Jansen Van Vuren et al. 2017), and so on, and speculated that they are potential vectors for pathogen transmission.

The *Nycteribia parvula* belongs to the genus Nycteribia of the subfamily Nycteribiinae of the family Nycteribiidae (Porter et al. 2022). Currently, studies on this species have focused on morphological description, life history, host distribution, and disease transmission, with less research on the mitochondrial genome. With its maternal inheritance, relatively high mutation rates, and absence of recombination, the mitochondrial genome has been widely utilized as genetic markers in molecular phylogenetic investigations (Yang et al. 2022). Therefore, the complete mitochondrial genome of *N. parvula* was sequenced and annotated for the first time. This adds to the molecular data of species in the family Nycteribiidae. The phylogenetic analysis was performed using some sequences of the superfamily Hippoboscoidea.

## Materials

### Sample collection

The specimens of *N. parvula* which were captured on the body surface of *Miniopterus fuliginosus* were collected from Binchuan county (100.58′E, 25.83′N), Yunan province, China, on 15 July 2022 and then identified by JT Yang. The specimens were preserved in 95% ethanol and kept in a −20 °C refrigerator in the Institute of Pathogens and Vectors, Dali University (URL: https://www.dali.edu.cn/kxyj/yjs/1611.htm, contact person: Xiaobin Huang, huangxb633@nenu.edu.cn) under the voucher number: DLBC2022001.

#### Statement

Capture of *Miniopterus fuliginosus* (Mammalia: Chiroptera: Miniopteridae: Miniopterus) was performed in accordance with the guidelines and regulations approved by the Animal Ethics Committee at Dali University (name: Dali University Ethics Committee; approval ID: MECDU-202104-27).

## Methods

### DNA extraction, mitogenome sequencing, and annotation

The total DNA was extracted from the insects’ whole body tissue using the Tissue DNA Kit (Omega, Norcross, GA). The Illumina Novoseq 6000 sequencing platform was used for sequencing, and the Illumina PE library was constructed. Then, this project used MitoZ 2.3 (https://doi.org/10.1101/489955) to assemble the mitochondrial genome (Meng et al. 2019), and the control region was verified by Sanger sequencing. The 13 PCGs of mitochondrial genome was annotated by MITOS (Donath et al. 2019) and tRNA and rRNA of the genome were annotated by GeSeq (Tillich et al. 2017) (using third party software tRNAscan-SE (Chan and Lowe 2019) and ARWEN (Laslett and Canbäck 2008)). Finally, the circular mitochondrial genome map was drawn using GENOMEVX (Conant and Wolfe 2008). The nucleotide sequences of *N. parvula* have been deposited in GenBank under accession number OP442519.

### Sequence analysis

**Figure 1.**
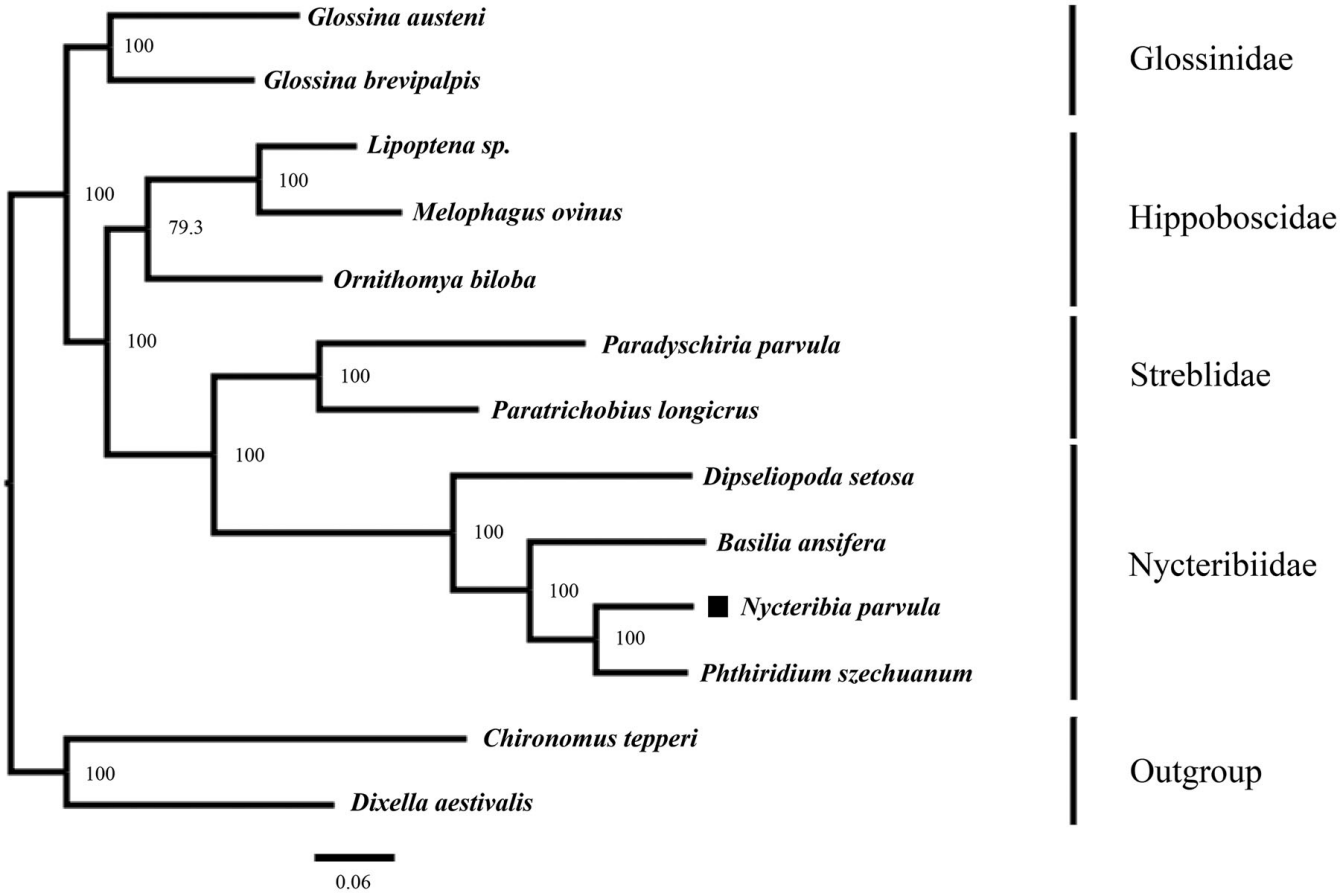
The maximum-likelihood phylogenetic tree of 11 species was inferred by IQ-TREE based on nucleotide sequence of 13 protein-coding genes. ‘■’ indicated newly sequenced data in this study. The vertical row indicates species of the same family.

The percentage codon usage of *N. parvula* was calculated using 'Geneious Prime 2020.1.2' software. *Chironomus tepperi* JN861749 (Beckenbach 2012) and *Dixella aestivalis* KT878382 (Briscoe et al. 2017) were selected as outgroups in the phylogenetic tree. The 13 protein-coding genes (PCGs) were aligned using the MAFFT (Katoh and Standley 2013). Based on nucleotide sequence of 13 PCGs, a phylogenetic tree between *N. parvula* and all of the sequences of the Superfamily Hippoboscoidea that have been published at the NCBI (a total of 10 species) was constructed using the IQ-TREE (Trifinopoulos et al. 2016) in PhyIoSuite software (Zhang et al. 2020) with the maximum-likelihood (ML) method (Figure 1), and using ModelFinder to determine GTR + F+I + G4 as the best model (Kalyaanamoorthy et al. 2017). Clade support was assessed using a nonparametric bootstrap with 1000 replicates. The phylogenetic tree was edited and visualized in the software FigTree.v1.4.4 (http://tree.bio.ed.ac.uk/software/figtree/). The sequences were used as follows: *Glossina austeni* MZ826152 (Porter et al. 2022), *Glossina brevipalpis* MZ826153 (Porter et al. 2022), *Lipoptena* sp MT679542 (Wang et al. 2021), *Melophagus ovinus* KX870852 (Liu et al. 2017), *Ornithomya biloba* MZ379837 (Li et al. 2022), *Paradyschiria parvula* MK896865 (Trevisan et al. 2019), *Paratrichobius longicrus* MK896866 (Trevisan et al. 2019), *Dipseliopoda setosa* MZ826151 (Porter et al. 2022), *Basilia ansifera* MZ826150 (Porter et al. 2022), and *Phthiridium szechuanum* OP459298 (unpublished).

## Results

### Morphological identification

**Figure 2.**
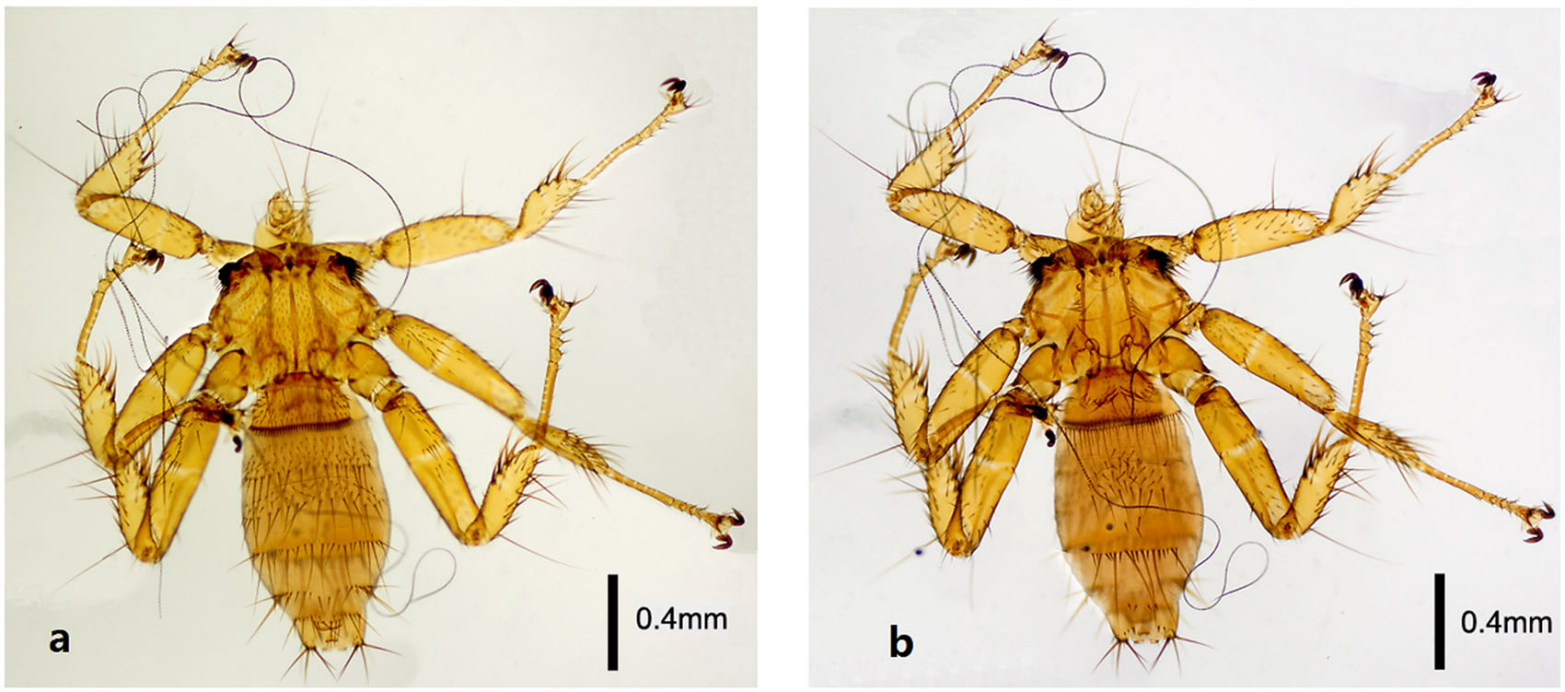
Morphological characteristics of female *N. parvula*: (a) the ventral of the *N. parvula*; (b) the dorsal of *N. parvula* (the photos were taken by JT Yang).

The *N. parvula* was determined under a Leica DM500 microscope (Leica, Wetzlar, Germany) using the keys provided by papers (Maa 1962). In morphological identification, the main points of *N. parvula* are the tergites, sternites, and female genital plates (Figure 2). The fore tibia is 2–2.5 times as long as it is wide. There are a few rows of moderately long setae near the back edge of tergite 1 on the female abdomen. Tergite 2 is very short with moderately long, thin setae at the hind margin and short hairs on the surface. Tergite 6 is broad with 5–6 long setae at the hind margin, which alternate groups of 1–3 pines. The anal segment is very short, wider than long. The dorsal genital plate is concave posteriorly with two setae.

### Characterization of the mitochondrial genome

**Figure 3.**
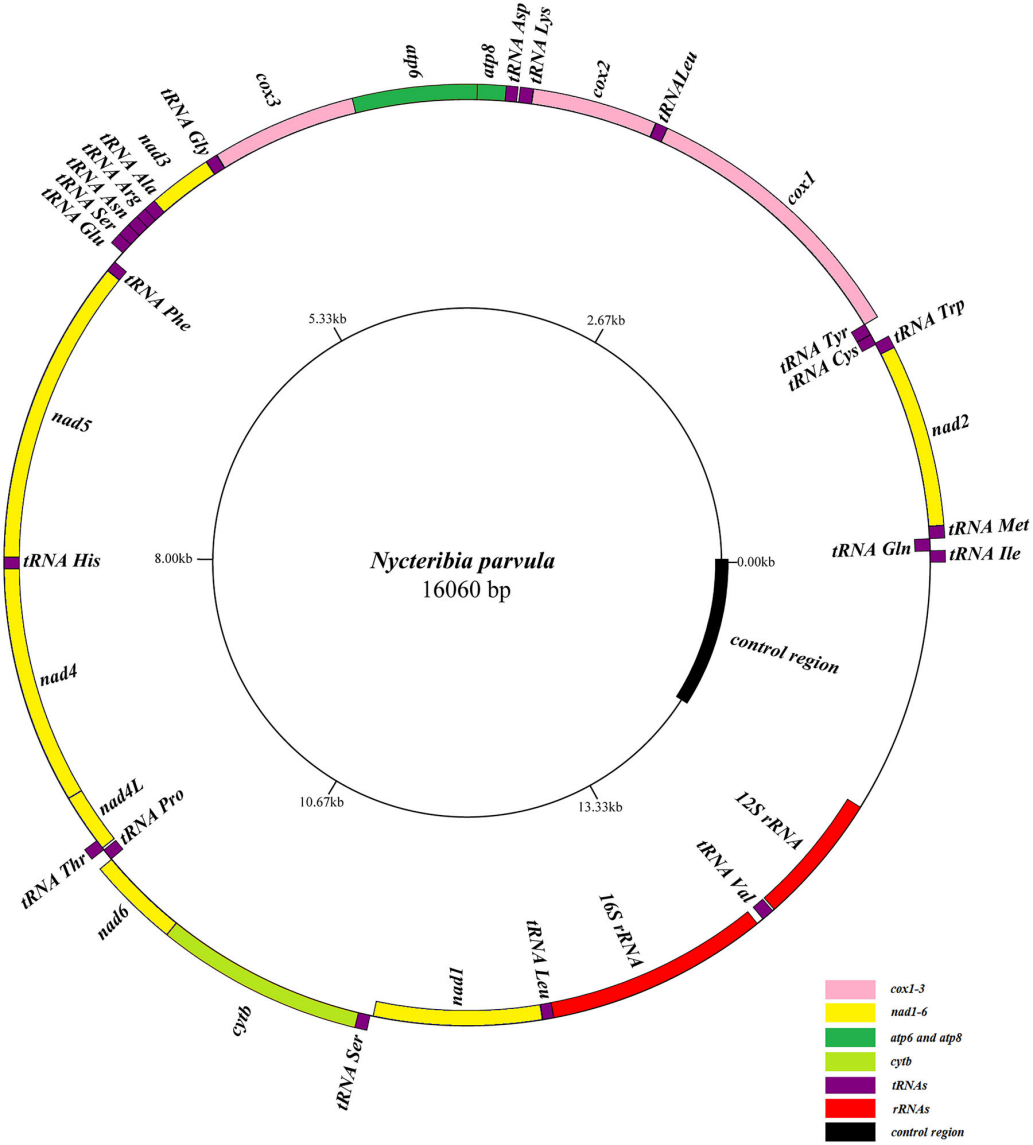
The circular mitochondrial genome map of *N. parvula.*

The complete mitochondrial genome of *N. parvula* (GenBank accession number: OP442519) is 16,060 bp and consists of 37 mitochondrial genes (13 PCGs, 22 transfer RNA genes, and two ribosomal RNA genes) and a control region (Figure 3). Gene content and gene order for the genomes were consistent with the predicted ancestral insect mitochondrial genome (Cameron 2014). The nucleotide contents of A, T, G, and C in the mitochondrial genome of *N. parvula* are respectively 40.86%, 42.19%, 6.51%, and 10.44%, and the proportion of AT content is 83.05%. The small subunit ribosomal RNA and large subunit ribosomal RNA of *N. parvula* had lengths of 741 bp and 1250 bp, respectively. Twenty-two tRNAs had various sizes, ranging from 61 bp (*tRNA Leu(uaa)*) to 71 bp (*tRNA Val*). Between the 12S rRNA and the *tRNA Ile*, a control region (1422 bp) with a 92.69% AT content was inserted. Except for three PCGs that start with TCG (*cox1*) and TTG (*nad1* and *nad5*), the other PCGs start with standard ATN codons (five ATT and five ATG). Except for two PCGs (*nad4* and *nad5*) that ended with incomplete stop codons T, the other 11 PCGs have complete stop codons (TAA or TAG).

### Phylogenetic analysis

Phylogenetic results indicate that the family Nycteribiidae is a monophyletic group, and the families Nycteribiidae and Streblidae were clustered on one branch with high nodal support values (BP > 90). Among them, *N. parvula* in the family Nycteribiidae has the closest affinity with *P. szechuanum*.

## Discussion and conclusions

In this study, we sequenced the complete mitochondrial genome of *N. parvula* for the first time and conducted a comprehensive phylogenetic analysis based on 13 PCGs of *N. parvula* and the other 10 species. The phylogenetic tree showed that the family Nycteribiidae is a monophyletic group and the species within the family clustered together with high confidence, indicating that the molecular classification of the family Nycteribiidae is consistent with the traditional morphological classification results. In conclusion, the complete mitochondrial genome of *N. parvula* was sequenced in this study, which can provide a reference for species identification and phylogenetic analysis.

## Data Availability

The genome sequence data that support the findings of this study are openly available in GenBank of NCBI at https://www.ncbi.nlm.nih.gov/, reference number OP442519. The associated BioProject, SRA, and Bio-Sample numbers are PRJNA883596, SRR21695883, and SAMN26036431, respectively.
